# Auditory attentional load modulates the temporal dynamics of audiovisual integration in older adults: An ERPs study

**DOI:** 10.3389/fnagi.2022.1007954

**Published:** 2022-10-17

**Authors:** Weiping Yang, Shengnan Li, Ao Guo, Zimo Li, Xiangfu Yang, Yanna Ren, Jiajia Yang, Jinglong Wu, Zhilin Zhang

**Affiliations:** ^1^Department of Psychology, Faculty of Education, Hubei University, Wuhan, China; ^2^Brain and Cognition Research Center (BCRC), Faculty of Education, Hubei University, Wuhan, China; ^3^Graduate School of Interdisciplinary Science and Engineering in Health Systems, Okayama University, Okayama, Japan; ^4^Department of Psychology, College of Humanities and Management, Guizhou University of Traditional Chinese Medicine, Guiyang, China; ^5^Applied Brain Science Lab, Faculty of Interdisciplinary Science and Engineering in Health Systems, Okayama University, Okayama, Japan; ^6^Research Center for Medical Artificial Intelligence, Shenzhen Institute of Advanced Technology, Chinese Academy of Science, Shenzhen, China

**Keywords:** audiovisual integration, aging, auditory attentional load, rapid serial auditory presentation (RSAP), ERPs

## Abstract

As older adults experience degenerations in perceptual ability, it is important to gain perception from audiovisual integration. Due to attending to one or more auditory stimuli, performing other tasks is a common challenge for older adults in everyday life. Therefore, it is necessary to probe the effects of auditory attentional load on audiovisual integration in older adults. The present study used event-related potentials (ERPs) and a dual-task paradigm [Go / No-go task + rapid serial auditory presentation (RSAP) task] to investigate the temporal dynamics of audiovisual integration. Behavioral results showed that both older and younger adults responded faster and with higher accuracy to audiovisual stimuli than to either visual or auditory stimuli alone. ERPs revealed weaker audiovisual integration under the no-attentional auditory load condition at the earlier processing stages and, conversely, stronger integration in the late stages. Moreover, audiovisual integration was greater in older adults than in younger adults at the following time intervals: 60–90, 140–210, and 430–530 ms. Notably, only under the low load condition in the time interval of 140–210 ms, we did find that the audiovisual integration of older adults was significantly greater than that of younger adults. These results delineate the temporal dynamics of the interactions with auditory attentional load and audiovisual integration in aging, suggesting that modulation of auditory attentional load affects audiovisual integration, enhancing it in older adults.

## Introduction

In daily life, individuals are constantly exposed to the information from different sensory sources, such as visual and auditory information. Previous studies have found that bimodal audiovisual stimuli can be discriminated or detected more rapidly and accurately than visual or auditory stimuli presented alone, and cross-modality processing advantages were evident (Stefanics et al., 2005; Ren et al., 2018). This facilitative effect is called audiovisual integration (AVI) (Parker and Robinson, 2018; Yang et al., 2021). Previous research has demonstrated that audiovisual integration is influenced by many factors, including attention (Talsma, 2015; Lunn et al., 2019).

Audiovisual integration helps us perceive information better, which in turn is intimately linked to attention. The interplay between audiovisual integration and attention allows us to dynamically select and process sensory signals that are relevant to behavior (Mishra and Gazzaley, 2012). Early studies have found that the audiovisual integration effect can only occur under attended conditions and that attention can boost it (Talsma and Woldorff, 2005). However, attentional resources are limited, according to the load theory proposed by Lavie et al.; if the attentional demand proves to be higher for one task, fewer attentional resources are available for other tasks (Lavie and Fox, 2000; Lavie and Fockert, 2003; Lavie and De Fockert, 2005). The number of resources involved in attention processing that are required is defined as the attention load (Cochrane et al., 2020). An increasing number of studies have explored the effect of attentional load on the processing of audiovisual integration. Some behavioral studies have found that audiovisual integration was attenuated by attentional load (Alsius et al., 2005, 2007). Further evidence by electroencephalogram (EEG) revealed the early auditory ERP components, and the N1 and P2 peaks present earlier in response to audiovisual stimuli relative to auditory stimuli in a single task. In addition, the latency decrement was reduced when attention was manipulated by dual tasks (Alsius et al., 2014). These studies suggested that the audiovisual integration effect was impaired by attentional load. Nevertheless, some researchers have discovered that visual and auditory sensory processes might handle attentional demands in a different way (Murphy et al., 2013). The spatial selectivity of vision provides a mechanism by which to focus processing capacity relatively on selected parts of sensory input. Visual attentional load has also been suggested to alter the spatial focus of attention (Murphy et al., 2016). In contrast to visual attention, hearing attention acts as an “early warning system,” which can monitor the environment from all directions rather than other spatially restricted modalities (Dalton and Lavie, 2004; Murphy et al., 2017). The auditory attention load focuses more attention on a continuous stream of auditory stimuli (Shinn-Cunningham, 2008). Indeed, the handling of seemingly irrelevant sounds may be beneficial to our survival from an evolutionary perspective (e.g., allowing us to detect the sound of a predator approaching from behind), which supports evidence that irrelevant auditory stimuli might be useful (e.g., as alerts) during complicated situations (Murphy et al., 2013). Individuals are more likely to be “deaf” to the voices when they are engaged in high auditory load tasks; for example, when listening intently to a radio while driving, a person may not hear other important sounds (Fairnie et al., 2016). These cases provided evidence of the meaningfulness of the existence of auditory load. Therefore, an interesting question is whether and to what extent bottom-up auditory attentional load affects audiovisual integration. According to load theory (Lavie, 1995; Lavie and De Fockert, 2005), attentional resources are limited. Perceptual processing can take place automatically and in parallel when attentional resources are not exceeded, whereas irrelevant stimuli cannot be processed when processing capacity is exceeded. In addition, attentional resources can be depleted to varying degrees due to the interference of irrelevant stimuli under high-load conditions. For this reason, we anticipated that audiovisual integration would be affected by attentional load and that greater audiovisual integration would be observed when attentional resources were sufficient.

Additionally, population aging is a global social issue and is one of the main challenges of the future, at least for the next few decades. Age-related declines in sensory systems in older adults have been observed. Most obviously, visual acuity tends to decrease, and hearing thresholds generally increase with age (Chou et al., 2016; Jayakody et al., 2018). However, much research on audiovisual integration has shown that older adults received greater benefits from audiovisual gain than younger adults, and an improvement in behavioral performance was observed (Laurienti et al., 2006; Peiffer et al., 2007; Winneke and Phillips, 2011; Sekiyama et al., 2014; Setti et al., 2014). Similarly, researchers using event-related potentials (ERPs) revealed significant multisensory P2 in the central and frontocentral regions, which indicated that older adults show greater audiovisual facilitation effects than younger adults in spatial discrimination processes (Zou et al., 2017). Other studies reported that activity in the posterior parietal and medial prefrontal regions was stronger in older adults than in younger adults when cross-modal stimuli were onset 100 ms later, and the network of posterior parietal and medial prefrontal activity underlies the integrated response in older adults (Diaconescu et al., 2013). Furthermore, other studies further reported that older adults integrated under a wider range of stimulus conditions than younger adults, and the temporal window of audiovisual integration for older adults was wider than that of younger adults (Zhou et al., 2020). These studies highlight that older adults exhibit greater integration of audiovisual stimuli than younger adults and predict that audiovisual integration may be an effective compensatory mechanism (Laurienti et al., 2006; de Boer-Schellekens and Vroomen, 2014; Diaz and Yalcinbas, 2021). However, considering that aging is associated with age-related decline in attentional resources (Blanchet, 2015), attentional load greatly affects the detection of stimuli in older adults. Therefore, one critical question is the extent to which audiovisual integration of older adults was affected by the attentional load. Recent studies have found that visual attentional load reduces the audiovisual integration effect, and that the audiovisual integration effect increases in older adults but is delayed. In addition, a shift in AVI oscillation from anterior to posterior regions in older adults is an adaptive mechanism (Ren et al., 2020, 2021b). Researchers have also explored the effect of auditory attentional load on audiovisual integration from a behavioral perspective. In this study, an audiovisual discrimination task was conducted with a rapid serial auditory presentation (RSAP) task competing for attentional resources. Consistent with the results of a previous study, the analysis of the race model demonstrated that audiovisual integration was decreased and delayed by auditory attentional load. Meanwhile, the audiovisual integration effect of older adults was lower and more delayed than in younger adults under all auditory attentional loads (Ren et al., 2021a). However, the temporal dynamics by which audiovisual integration interacts with auditory attentional load in older adults are not yet clear.

To address this question, the present study used ERP to compare audiovisual integration under different auditory attentional load conditions in older and younger adults. In the current study, participants performed a Go/No-go task, which was used to gauge the effects on audiovisual integration. The RSAP task was designed to modulate attentional demands during the Go/No-go task, and the experiment was divided into 4 blocks (no-, low-, medium-, and high-attentional load). The compensation-related utilization of neural circuits hypothesis (Reuter-Lorenz and Cappell, 2008) highlights that the brain needs to use more cognitive resources at a time when older adults have less neural resources to achieve similar levels of cognition as younger adults. Considering the compensation mechanisms of the older adults, we can speculate that the audiovisual integration benefits are greater for older adults than for younger adults under different auditory attentional loads.

## Methods

### Participants

In total, 38 younger adults and 36 older adults were recruited for the study. Before the experiment, the Mini-Mental State Evaluation Scale (MMSE) was used to initially screen the degree of cognitive impairment and the presence or absence of dementia in older adults and to evaluate their cognitive function. In total, two older adults with an MMSE score lower than 27 points (a score of <27 was used as an exclusion criterion) (Folstein et al., 1975; Kukull et al., 1994) were excluded. In total, three older adults and four younger adults were excluded from the analysis due to poor performance in ERP data collection (loss of >70% of epochs). In addition, two adults were excluded from further analyses because they did not complete this experiment. Additionally, four younger adults who had <50%accuracy on single-visual, sing-hearing, and audiovisual targets were also excluded. Finally, the data of a subset of 30 younger adults and 29 older adults were used for further analyses. Demographic characteristics and group differences for the final sample are presented in Table 1. All participants had normal or corrected vision and normal hearing and were right-handed. In addition, the participants had no history of alcohol or drug abuse and no history of psychiatric or neurological disorders. Informed consent for the procedure was provided before the experiment was conducted, which had previously been approved by the Ethics Committee of Hubei University (no. 2019106). Moreover, all participants were naive to the study design and completed this experiment successfully. Participants were financially reimbursed for their time.

**Table 1:** Summary of participant characteristics.

	**Younger**	**Older**
N	30	29
Mean age (stdev)	20.1(1.3)	63.8 (4.2)
Sex (M/F)	16:14	12:17
Years of education (stdev)	14.3 (0.7)	11.4 (1.6)
Score of the MMSE (stdev)	–	29.4 (0.9)

### Task

#### The Go/No-go task

**Figure 1:**
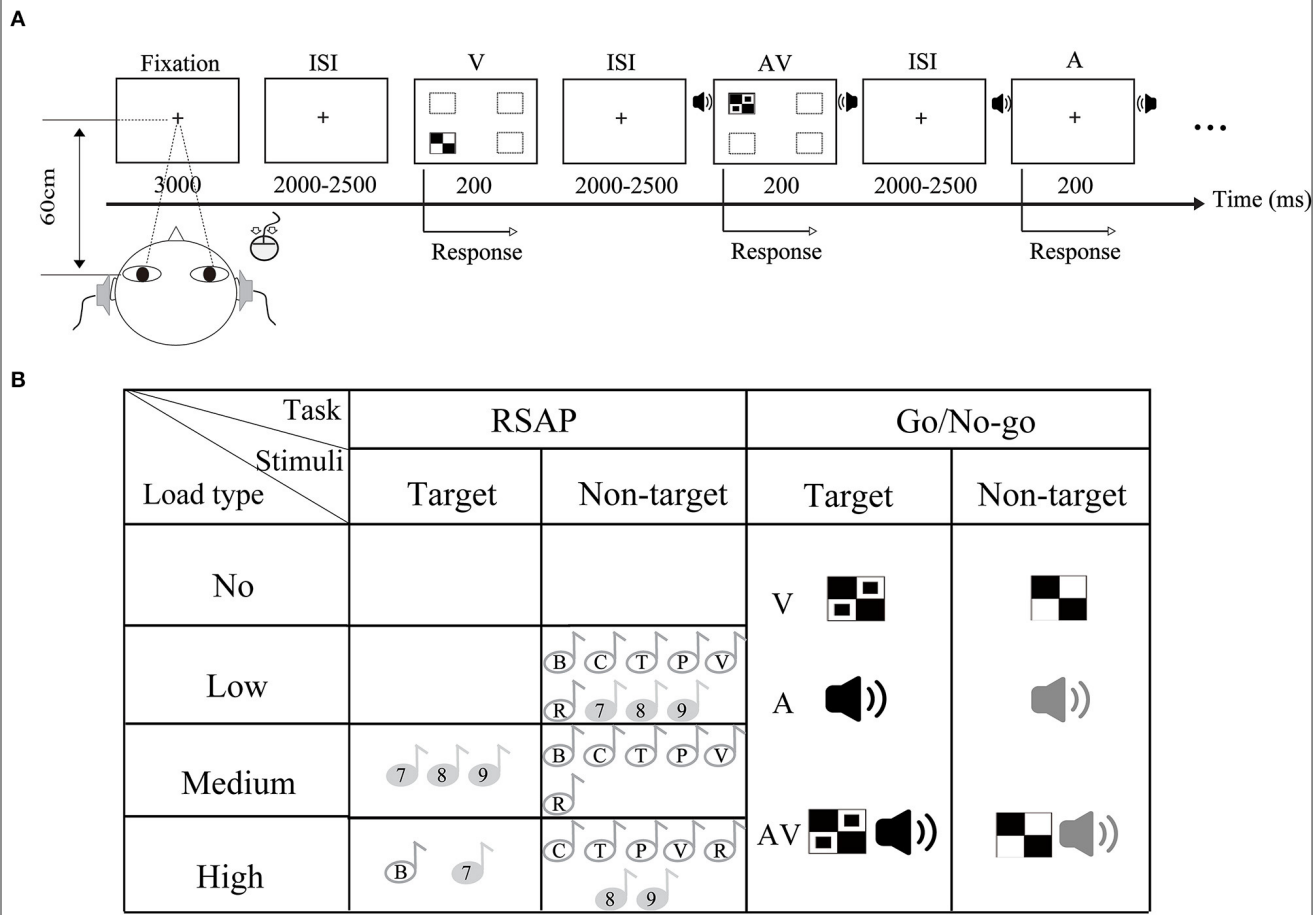
Schematic illustration of experimental paradigm and material. **(A)** Stimuli of the Go/No-go task were randomly presented in visual (V), auditory (A), and audiovisual modalities (AV). In this experiment, the RSAP task was purposively controlled to be present. **(A)** Showed the No_load condition. **(B)** Representation of the dual task stimuli for the different attentional load conditions. This figure illustrates a load manipulation in which the task varies.

All stimuli were divided into two types: target stimuli and non-target stimuli. The visual non-target stimulus was a checkerboard image (Black-and-White checkerboard, 52 mm × 52 mm, with a visual angle of 5°). The visual target stimulus was a B/W checkerboard image with two black dots contained within each white checkerboard (Figure 1A). The auditory target stimulus was white noise at 60 dB, and the auditory non-target stimulus was a 1,000 Hz sinusoidal tone (rise and fall time at 10 ms). In addition, the audiovisual target stimuli consisted of the simultaneous presentation of the visual and auditory target stimuli. The audiovisual non-target stimuli were prepared along the same principle as the target stimuli. The visual stimuli were presented for 200 ms with a 12-degree visual angle in four directions of the computer monitor: lower left, upper left, lower right, and upper right. The auditory stimuli (A) were presented at 200 ms through headphones at a sound-pressure level (SPL) of approximately 60 dB. The ratio of the target stimulus to the non-target stimulus was 4:1 for each stimulus type in the total set of stimuli.

#### The RSAP task

This task presents a stream of stimuli to the participant sequentially and at a constant time and consists of 9 characters taken from 6 letters (B, C, P, T, V, and R) and 3 digits (7, 8, and 9). Each character was presented 10 times for 200 ms, and the interstimulus interval ranged from 2,000 to 2,500 ms. These stimuli were presented left and right through the headset. All stimuli were delivered in a pseudorandomized order that was balanced across participants and conditions.

### Procedure

The experiment was performed in a dimly lit and sound-attenuated room. The experiment was divided into 4 blocks (no, low-, medium-, and high-attentional loads), as illustrated in Figure 1B. The order of blocks was randomized across participants. Each block consisted of 20 trials for each target stimulus type (A, V, and AV) and 80 trials for each non-target stimulus type (A, V, and AV) in the Go/No-go task and was accompanied by 90 trials of random characters. There were 300 trials for the no-attentional load condition, 345 trials for the other conditions, and a total of 1,335 trials for the experiment. Including breaks, the duration of the experiment was ~90 min.

Participants performed 20 practice trials for each load condition before the start of the experiment. As shown in Figure 1, each trial started with a fixation cross displayed in the center of the screen for 3,000 ms. Then, the auditory and visual stimuli of the Go/No-go task were presented separately or simultaneously for 200 ms. At the same time, the RSAP task was manipulated to occur, i.e., the character of the RSAP task was present at low-, medium-, and high-attentional load conditions. The Go/No-go task and the RSAP task occurred simultaneously. This was followed by the display of a fixation cross for 2,000–2,500 ms, during which participants were asked to point to by pressing a button. In the no-load condition, participants were asked to focus on target stimuli presented by different modalities of the Go/No-go task and responded to the target stimuli by pressing the left mouse button. In the low-attentional load condition, participants were asked to monitor the target stimuli in the Go/No-go task, irrespective of the simultaneous presentation of auditory distractors from the RASP task. In the medium-attentional load condition, the participants were instructed to respond to the target in the Go/No-go task and the target (7, 8, and 9) in the RSAP task. In the high-attentional load condition, the participants were asked to respond to the target in the Go/No-go task and to the target (B, 7) in the RSAP task. During the medium- and high-attentional load blocks, participants were instructed to perform both tasks simultaneously to the best of their abilities, i.e., one task was not prioritized over the other. No response was required for non-targets in the whole experiment. In general, the Go/No-go task was equivalent in four attentional load blocks; however, the reactive mode of the RSAP task was purposively controlled due to the experimental goal.

### Apparatus

Stimulus presentation and recording of participants' responses were implemented using the software E-Prime 2.0 (Psychology Software Tools, Inc., Pittsburgh, PA, USA). Visual stimuli were controlled and presented on a 15.6-inch visible LCD screen with a 1,024 × 768 pixel resolution and 100 Hz refresh rate. Auditory stimuli were controlled by a USB audio interface and delivered through in-ear headphones.

The behavioral data and electroencephalogram (EEG) were recorded simultaneously. An EEG system (BrainAmp MR plus, Gilching, Germany) was used to capture EEG signals through an electrode cap with 32 electrodes (Easy-cap, Herrsching Breitbrunn, Germany) and a matching EEG amplifier (Gilching, Germany). The electrodes are arranged according to the International 10–20 system based on the position of the head (forehead), with all signals directed to the left and right earlobes. Vertical eye movement and blinking were measured by capturing EOG data from an electrode located approximately 1 cm below the subjects' left eye. Horizontal eye movement was measured by obtaining EOG signals from an electrode placed approximately 1 cm (HEOG) outside the subject's right eye. All signals refer to FCZ. Standard stimuli were used to induce event-related potentials for further analysis. During the experiment, the impedance of all electrodes was kept below 5 kΩ. The original signal is digitized using a sampling rate of 500 Hz, and all data are stored digitally for offline analysis.

### Data analysis

**Figure 2:**
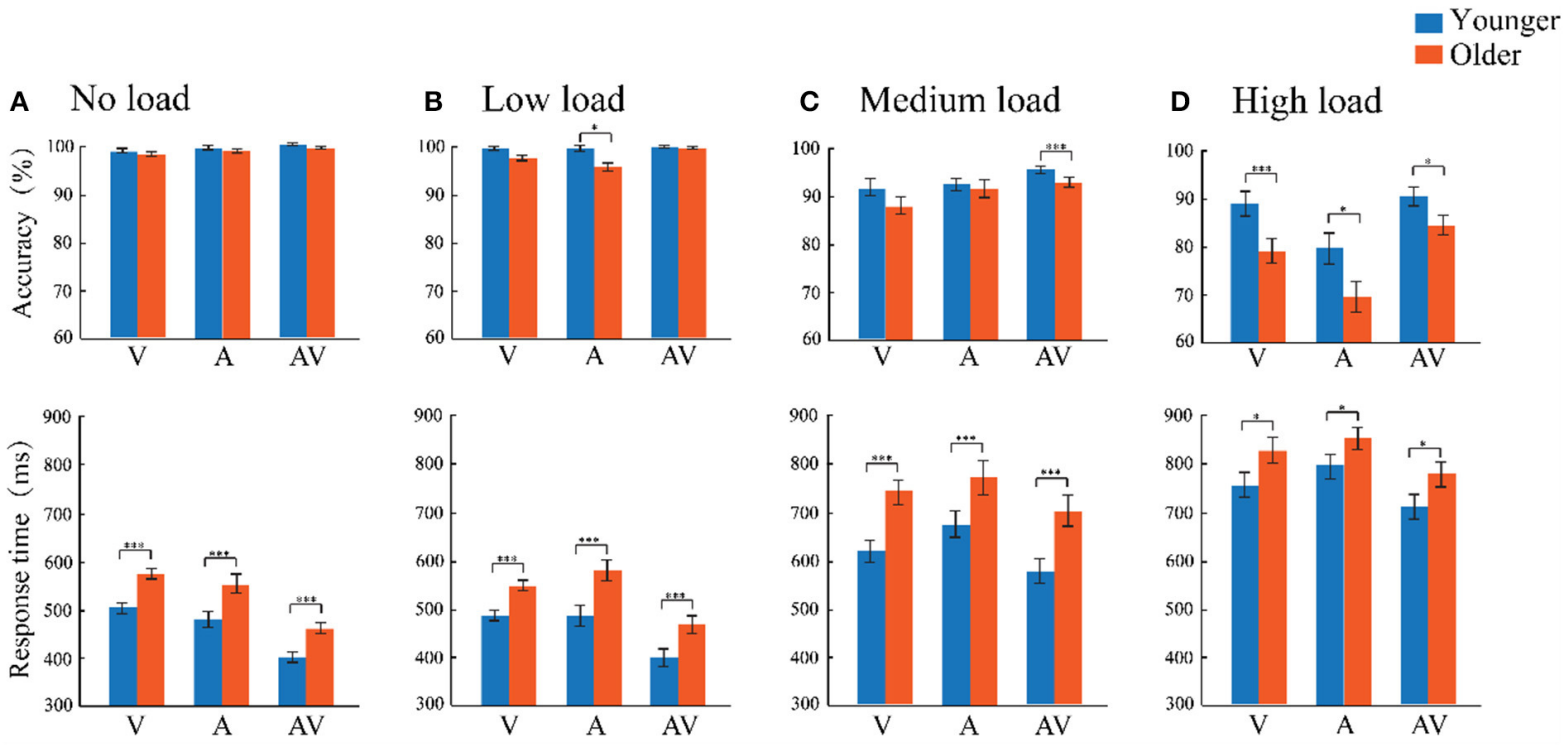
**(A–D)** Comparison of the accuracy and response time between the older adults (orange) and younger adults (blue) for the three stimulus types (A, V, and AV) under both the no-, low-, medium-, and high-attentional load conditions. The standard error of the mean (SEM) was also presented. *p ≤ 0.05, ***p ≤ 0.001.

Accuracy (ACC) and response time (RT) for target stimuli were computed separately for each stimulus type in four attentional load conditions. Additionally, data exceeding the average response ± 3 standard deviations were eliminated, and the final amount of data deleted accounted for 1.56% of the total data. The accuracy and response time are shown in Figure 2. A mixed-factors ANOVA of 2 (age: older adults and younger adults) × 4 (load level: no, low, medium, and high load) × 3 (modality: visual, auditory, and audiovisual) was conducted for accuracy and response time. The Greenhouse–Geisser Epsilon correction was applied to adjust the degrees of freedom of the F ratios as necessary.

Event-related potential data preprocessing was performed using Brain Vision Analyser software (version 1.05, Brain Products GmbH, Munich, Bavaria, Germany). Offline analysis was used to rereference all electrodes with signals from bilateral mastoid electrodes (TP9/TP10). The standard stimuli were used to elicit the ERPs for further analysis. The original EEG signals were filtered by bandpass in the range of 0.01–60 Hz, and the standard stimuli were segmentally processed by visual marks, auditory marks, and audiovisual marks. Data from a total of 450 time points were selected for analysis from 100 ms before the start of the stimulus to 800 ms after the start of the stimulus. Baseline correction was then performed with the signal measured relative to the −100 to 0 ms before the stimulus began. EEG activity and other motion artifacts exceeding ±100 μV were eliminated. The data for each stimulus type were then averaged overall. In addition, bandpass filtering was carried out again at the standard of 0.3–30 Hz, and baseline correction was performed at −100 to 0 ms. Overall average data for each stimulus type in each electrode for each age group were obtained under different loads. For the collapsed no-attentional load condition, the average number of non-artifact epochs for each subject is 74.4. The average number of non-artifact epochs for each subject for the low-, medium-, and high-attentional load conditions was 60.8, 56.4, and 50.5, respectively, which is an ideal number of epochs.

**Figure 3:**
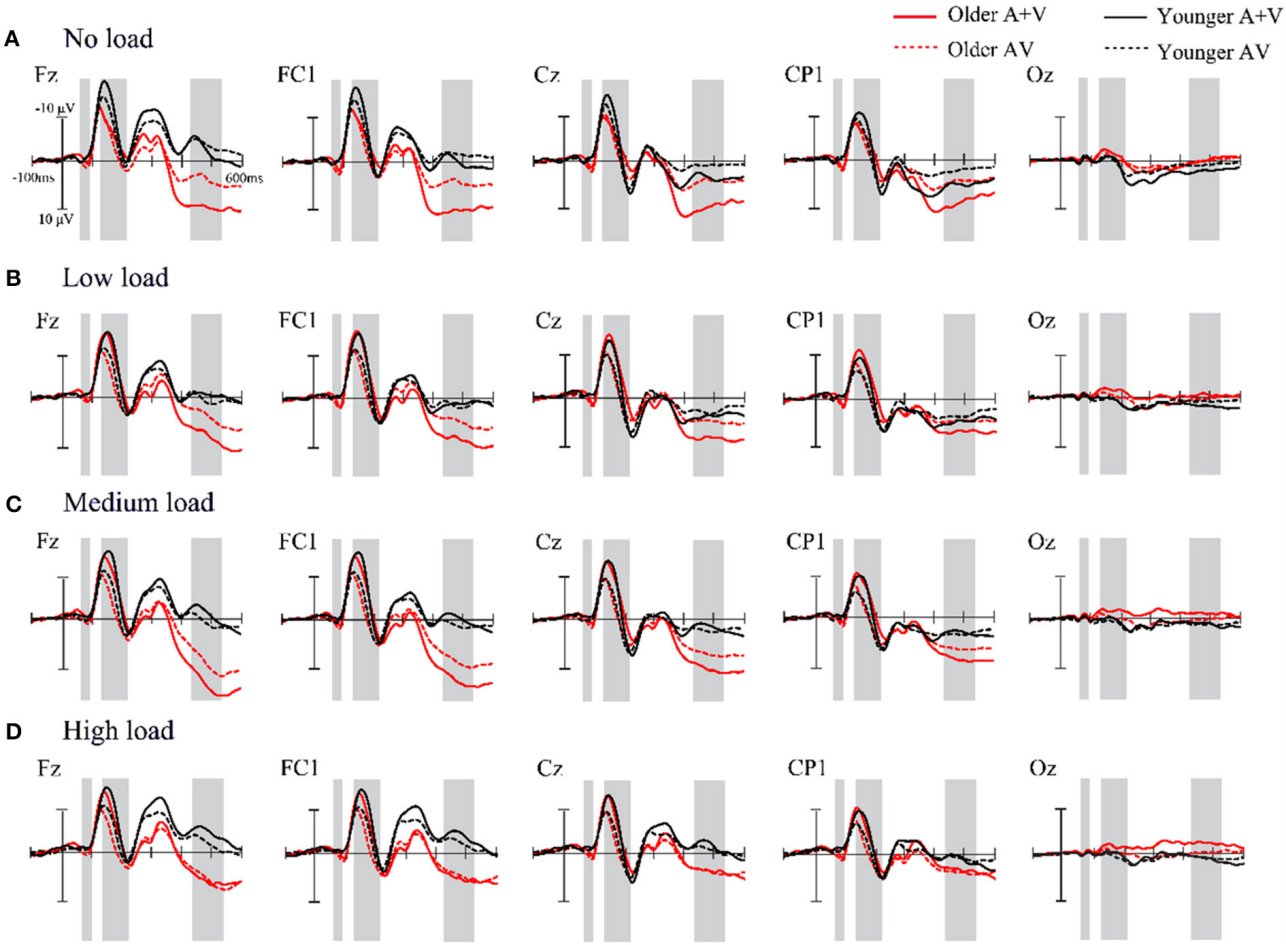
**(A–D)** Comparison of the sum of the event-related potentials of the unimodal stimuli (A + V) and the event-related potentials of the bimodal stimuli (AV) for a time interval of 0–600 ms in the representative electrodes under the different load conditions. Shaded areas mark time intervals where a significant difference is found.

In line with previous studies, audiovisual integration can be evaluated by the difference wave, ERP[AV – (A + V)], which was acquired by subtracting the amplitude of linear summation of respective unisensory constituents from the amplitude of audiovisual stimuli (Walden et al., 1993; Senkowski et al., 2011; Ren et al., 2018; Yang et al., 2021). The statistical analysis of audiovisual interactions focused on the three distinct main response intervals shown as shaded areas in Figure 3: 60–90 ms, 140–210 ms, and 430–530 ms after stimulus onset. This was acquired using pointwise and running *t*-tests for electrodes (two-tailed) comparing 0 under each condition to the difference wave from 0 to 600 ms. Audiovisual integration occurred when at least 12 continuous data points met the α criteria of 0.05 (12 data points = 24 ms at a 500 Hz digitization rate). Based on the topographical response pattern and previous studies (Ren et al., 2018; Yang et al., 2021), the regions of interest (ROIs) were selected for further analysis, including the frontal cortex region: F3, F7, Fz, F4, and F8; the frontal central cortex region: FC1, FC2, FC5, and FC6; the central cortex region: C3, C4, and CZ; the centroparietal region: CP5, CP1, CP2, and CP6; and the occipital lobe region: O1, OZ, and O2. The statistical analysis was carried out on amplitude averages across time intervals (60–90, 140–210, and 430–530 ms) within each of the five ROIs using a mixed-factors ANOVA. All statistical analyses were performed using SPSS 21.0 software, and Greenhouse–Geisser Epsilon correction was applied to adjust the degrees of freedom.

## Results

### Behavioral results

The accuracy was analyzed using a 2 (age: older adults and younger adults) × 4 (attentional load: no, low, medium, and high load) × 3 (modality: auditory, visual, and audiovisual) mixed-factors ANOVA with age set as the between-subject factor and attentional load and modality set as within-subject factors. The results showed that the main effect of age was significant, [*F*_(1, 57)_ = 20.422, *p* < 0.001, $\eta_{\text{p}}^{2}$ = 0.264]. The accuracy rate of younger adults (94.80%) was significantly higher than that of older adults (91.10%). The main effect of load level was observed, [*F*_(3, 171)_ = 116.430, *p* < 0.001, $\eta_{\text{p}}^{2}$ = 0.671]. Pairwise comparisons revealed the highest accuracy in the no- (98.90%) and low- (98.70%, *p* = 0.626) attentional load conditions compared with medium- (*p* < 0.001) and high-attentional load conditions (*p* < 0.001), which confirmed that the high-attentional load task was more demanding. The main effect of modality was significant, [*F*_(2, 114)_ = 13.141, *p* < 0.001, $\eta_{\text{p}}^{2}$ = 0.187], with the accuracy for the audiovisual stimulus (95.20%) being significantly higher than that of visual-only (93.10%, *p* = 0.026) and auditory-only stimuli (90.70%, *p* = 0.004), which showed the facilitating effect of audiovisual processing. In addition, the accuracy of the visual stimulus was significantly higher than that of the auditory stimulus (*p* < 0.001). In addition, the interaction of attentional load × age was significant [*F*_(3, 171)_ = 116.342, *p* < 0.001, $\eta_{\text{p}}^{2}$ = 0.671]. The *post-hoc* analysis using pairwise comparison with Bonferroni correction for attentional load showed that the accuracy of the older and younger adults under the no-attentional load and low-attentional load was significantly higher than that under the medium- and high-attentional loads (all *p* ≤ 0.001), but there was no significant difference between no- and low-load conditions (*p* = 0.100). The *post-hoc* analysis using pairwise comparison with Bonferroni correction for age revealed that the accuracy of younger adults was significantly higher than that of older adults in the low-, medium-, and high-attentional load conditions (all *p* ≤ 0.032), but there was no age difference in the no-attentional load condition (*p* = 0.162).

Similarly, a 2 (age: older adults and younger adults) × 4 (attentional load: no, low, medium, and high load) × 3 (modality: auditory, visual, and audiovisual) mixed-factors ANOVA was conducted for the response time. Analysis showed a significant main effect for age [*F*_(1, 57)_ = 21.781, *p* < 0.001, $\eta_{\text{p}}^{2}$ = 0.276], and the analysis revealed faster responses in younger adults (M = 573 ms, SE = 12 ms) compared to older adults (M = 653 ms, SE = 12 ms). There were significant attentional load main effects [*F*_(3, 171)_ = 464.734, *p* < 0.001, $\eta_{\text{p}}^{2}$ = 0.819)], showing that responses for the low- (M = 492 ms, SE = 8 ms) and no-attentional load (M = 493 ms, SE = 7 ms) conditions were significantly faster than those for medium- (M = 683 ms, SE = 12 ms, *p* < 0.001) and high-attentional loads (M = 784 ms, SE = 14 ms, *p* < 0.001). In addition, there was no difference in the no- and low-attentional load conditions (*p* = 0.100). Additionally, a significant modality main effect was also found [*F*_(2, 114)_ = 143.163, *p* < 0.001, $\eta_{\text{p}}^{2}$ = 0.715], showing that the response to audiovisual stimuli (M = 560 ms, SE = 10 ms) was significantly faster than those to visual (M = 630 ms, SE = 8 ms, *p* < 0.001) and auditory stimuli (M = 649 ms, SE = 10 ms, *p* < 0.001). In addition, visual stimuli were also significantly faster than auditory stimuli (*p* = 0.009) (AV _RT_ < V _RT_ < A _RT_). In addition, a significant age × attentional load interaction was observed [*F*(3, 171) = 3.644, *p* = 0.035, $\eta_{\text{p}}^{2}$ = 0.06]. To illustrate the age differences between the four attentional load conditions, an independent-sample *t-*tests (two-tailed) comparison with Bonferroni correction were separately conducted. The results showed a significant age difference between the no- [*t*_(57)_ = 5.060, *p* < 0.001], low- [*t*_(57)_ = 4.640, *p* < 0.001], medium- [*t*_(57)_ = 5.073, *p* < 0.001], and high- [*t*_(57)_ = 2.200, *p* = 0.032] attentional load conditions. These results revealed that the response by younger adults was faster than that by older adults across the four loading conditions.

### ERP results

#### Audiovisual integration for 60–90 ms

A mixed-factors ANOVA with 2 (age: older adults and younger adults) × 4 (attentional load: no, low, medium, and high) × 5 (ROI: frontal, frontocentral, central, centroparietal, and occipital) was performed to analyze the amplitude of the difference wave ERP(AV) – [ERP(A) + ERP(V)]. The results revealed a significant main effect of age [*F*_(1, 57)_ = 5.803, *p* = 0.019, $\eta_{\text{p}}^{2}$ = 0.092], suggesting that the amplitude of the difference wave for older adults was significantly greater (M =0.68 μV, SE = 1.15 μV) than for younger adults (M = 0.17 μV, SE = 1.50 μV). There was also a significant main effect of ROI [*F*_(4, 228)_ = 4.991, *p* = 0.011, $\eta_{\text{p}}^{2}$ = 0.081], with the amplitude of the difference wave for the amplitude of the frontal region (M = 0.62 μV, SE = 0.14 μV) being significantly greater than that in the frontocentral region (M = 0.43 μV, SE = 0.13 μV, *p* = 0.042). Additionally, no significant differences in other regions were found. In this time interval, the main effect of attentional load [*F*_(3, 171)_ = 1.091, *p* = 0.094, $\eta_{\text{p}}^{2}$ = 0.038] and interaction was not found (all *p* > 0.05).

#### Audiovisual integration for 140–210 ms

The integration effect was analyzed using similar ANOVAs with 2 × (age: older adults and younger adults) × 4 (attentional load: no, low, medium, and high) × 5 (ROI: frontal, frontocentral, central, centroparietal, and occipital). Analysis of these amplitudes showed that the effects of age were not significant [*F*_(1, 57)_ = 0.233, *p* = 0.632, $\eta_{\text{p}}^{2}$ = 0.004]. However, significant main effects of the attentional load [*F*_(3, 171)_ = 16.572, *p* < 0.001, $\eta_{\text{p}}^{2}$ = 0.225] were observed, with the amplitudes of the no load (M = 1.56 μV, SE = 0.30 μV) being significantly less than those of all other loads (low load: M = 3.15 μV, SE = 0.29 μV; medium load: M = 3.58 μV, SE = 0.25 μV; high load: M = 2.85 μV, SE = 0.25 μV, all *p* < 0.001). The amplitude in the medium load condition was significantly more positive than that in the high-load condition (*p* = 0.032), but there was no significant difference between the integrated amplitude of the low load and that of the medium- (*p* = 0.550) and high-load (*p* = 0.100) conditions. Additionally, the ROI main effect was also significant [*F*_(4, 228)_ = 67.011, *p* < 0.001, $\eta_{\text{p}}^{2}$ = 0.540], with the amplitudes of centroparietal (M = 2.74 μV, SE = 0.18 μV) and occipital (M = 0.82 μV, SE = 0.11 μV) regions being weaker than those of other regions (frontal region: M = 3.42 μV, SE = 0.29 μV; frontocentral region: M = 3.47 μV, SE = 0.29 μV; central region: M = 3.50 μV, SE = 0.26 μV, all pairwise *p* < 0.001). Furthermore, the centroparietal region was also significantly more positive than the occipital regions (*p* < 0.001). There are no differences between wave amplitudes in other regions.

**Figure 4:**
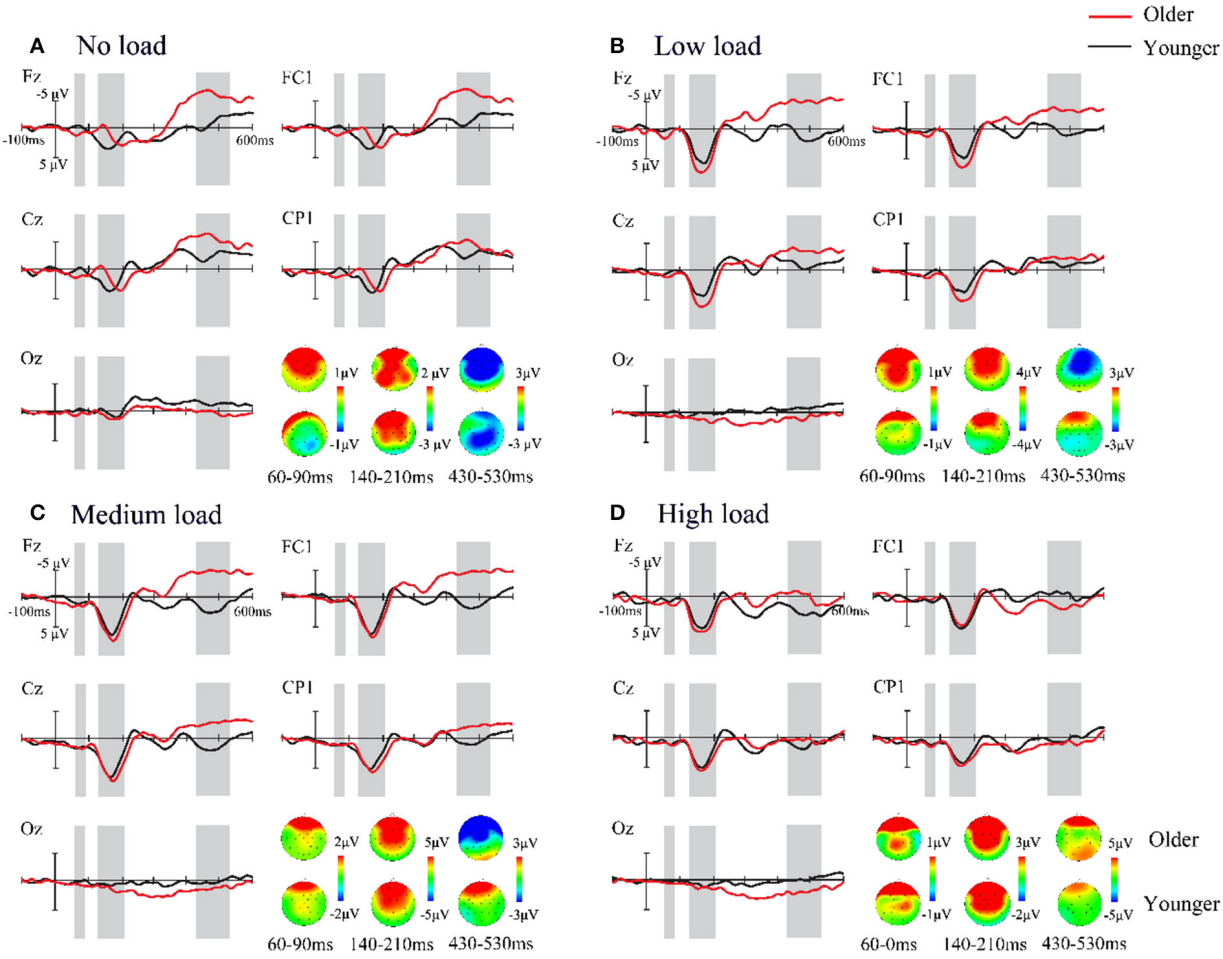
**(A–D)** Grand-average event-related potentials and topography maps in the four attentional load conditions. The difference wave, ERP [AV – (A + V)], is shown from 100 ms before to 600 ms after stimulus onset in the older (red) and younger (black) adults with the five ROIs, which shows the age difference in audiovisual integration processes. Time courses with significantly marked with a gray background. One representative electrode is shown separately per ROI.

Importantly, the interaction of attentional load and age was significant at this time interval. To illustrate whether there were age differences between the four attentional load conditions, we performed a simple effect analysis. The results demonstrated that for the low-load condition, there was a significant difference between older adults and younger adults, with the difference wave for the older adults (M = 3.77 μV, SE = 044 μV) being significantly greater than that for the younger adults (M =2.54 μV, SE = 0.39 μV, *p* = 0.005) (refer to Figure 4). For other attentional load conditions, no significant difference was found between the ages (all *p* > 0.05). Moreover, in terms of attentional load, we observed that for the older adults, the amplitudes of the no-load condition were less than those of the low-, medium-, and high-load conditions (all *p* < 0.001). For the younger adults, the medium load (M = 3.56 μV, SE = 0.36 μV) elicited a significantly greater difference wave compared to the no-load (M = 2.03 μV, SE = 0.42 μV, *p* = 0.008) and low-load (M = 2.54 μV, SE = 0.41 μV, *p* = 0.031) conditions.

#### Audiovisual integration for 430–530 ms

In this time interval, a 2 (age: older adults and younger adults) × 4 (attentional load: no, low, medium, and high) × 5 (ROI: frontal, frontocentral, central, centroparietal, and occipital) mixed-factors ANOVA was conducted. We observed the main effect of age [*F*_(1, 57)_ = 4.630, *p* = 0.036, $\eta_{\text{p}}^{2}$ = 0.075], and a larger amplitude for older adults (M = −2.58 μV, SE = 0.57 μV) than younger adults (M = −0.87 μV, SE = 0.56 μV) was found. The results also revealed a significant attentional load main effect [*F*_(3, 171)_ = 11.104, *p* < 0.001, $\eta_{\text{p}}^{2}$ = 0.163], indicating that the amplitude of no load (M = −3.75 μV, SE = 0.62 μV) was more negative than that of other attentional load conditions (low load: M = −1.61 μV, SE = 0.53 μV; medium load: M = −1.40 μV, SE = 0.55 μV; high load: M = −0.12 μV, SE = 0.52 μV, all *p* ≤ 0.021). However, there were no significant differences between the other load conditions (all pairwise *p* > 0.05). Additionally, a main effect of ROI was observed [*F*_(4, 228)_ = 10.894, *p* < 0.001, $\eta_{\text{p}}^{2}$ = 0.160]. The amplitude of the occipital region (M = −0.20 μV, SE = 0.19 μV) was significantly less than that of all other regions (all *p* ≤ 0.020). Moreover, there was a significant difference in the amplitude between the central regions and frontocentral regions (*p* = 0.02), but all other pairwise comparisons did not show significant differences (all *p* > 0.05). We did not observe the two-way interaction of age × attentional load at this time interval.

## Discussion

The main goal of the present study was to investigate the relationship between audiovisual integration and auditory attentional load in aging and to describe the temporal characteristics of audiovisual enhancement. Behavioral results showed that both older and younger adults responded faster and had higher hit rates to audiovisual stimuli than to visual and auditory stimuli alone and had poorer performance under high-attentional load demands. ERP revealed weaker audiovisual integration under the no-attentional auditory load condition at the earlier processing stages and, conversely, stronger integration in the late stages. Furthermore, older adults displayed enhanced audiovisual integration compared with younger adults at the time intervals of 60–90, 430–530, and 140–210 ms. Notably, only under the low-load condition in the time interval of 140–210 ms, we did find the audiovisual integration of older adults to be significantly greater than that of younger adults.

### Reversal of auditory attentional load on the regulation of audiovisual integration

In this study, weaker audiovisual integration under the no-attentional auditory load condition at the earlier processing stages was observed. The effects of the auditory load task on the processing of audiovisual integration are inconsistent with our hypotheses. This might be due to the difference in audiovisual integration at the early and late stages. In accordance with the time window of integration (TWI) model, audiovisual integration consists of two stages: early stages of perception of unimodal information and late stages of integrated information processing (Diederich and Colonius, 2004; Diedericha et al., 2008). In line with the load theory by Lavie et al. (2014), there is a top-down selection of conscious engagement within the early stages, and awareness will depend on the level of attentional load of the attended processing. This provided one possible interpretation that individuals pay more conscious attention to the target information in Go/No-go tasks when they deal with the higher attentional load. Conversely, less conscious input for the audiovisual target stimuli under the no-load condition weakened audiovisual integration. Another possible reason for the reduced earlier audiovisual integration under the no-attentional load condition might also be attributed to the decline in arousal levels. According to the theory of resource limitation of attention, the level of arousal determines the number of attentional resources available (Kahneman, 1973). In the no-load condition, the participants attended to the stimuli of the Go-NoGo task. In comparison with low-, medium- and high-load conditions, individuals not only need to attend to the stimuli of the Go-NoGo task but also pay attention to stimuli of the RSAP task. This means that individuals have decreased levels of arousal in the no-load condition compared with low-, medium-, and high-load conditions. Thus, audiovisual integration was weaker in the early stages of perception of unimodal information under load conditions.

In contrast, greater audiovisual integration under the no-attentional load condition at a late stage was found. Since the individual only needs to respond to the target stimulus in the Go/No-go task under no-load conditions, following the load theory, individuals have sufficient attentional resources to process audiovisual information in lower attentional load conditions (Lavie and Tsal, 1994; Lavie, 1995). Additionally, Lavie noted that there are two types of attentional mechanisms: early perceptual selection and late cognitive control. Early attentional selection is a passive selection process that works mainly under high perceptual load, whereas cognitive control acts as a late active control process that works mainly under low perceptual load (Lavie et al., 2004). Attention modulation of audiovisual integration in the later stages of the process has also been widely reported (Talsma and Woldorff, 2005; Koelewijn et al., 2010; Altieri and Townsend, 2011; Gibney et al., 2017). The presence of adequate resources and the role of top-down attention could be the fundamental reason for greater audiovisual integration at the later processing stages under no-attentional load. In general, our study is in good accordance with previous studies, which showed that top-down attention is engaged in the multiple stages of audiovisual integration and modulates audiovisual integration processes (Talsma et al., 2007; Beck and Kastner, 2009; Koelewijn et al., 2010; Talsma, 2015).

### Audiovisual integration enhancement in older adults

Our results are in line with previous studies showing that older adults seem to have more gains in audiovisual integration than younger adults (Laurienti et al., 2006; Peiffer et al., 2007; Diedericha et al., 2008; Winneke and Phillips, 2011; Mozolic et al., 2012; Diaconescu et al., 2013; Parker and Robinson, 2018). These results are in agreement with our original hypothesis. While age-related declines in perceptual abilities have been previously demonstrated, researchers have found that older adults exhibit greater functional connectivity and higher network efficiency in theta and alpha bands than younger adults (Wang et al., 2017, 2018). It was also found that older adults activate higher levels of brain activity than younger adults, and additional prefrontal cortex (PFC) and frontoparietal activity boosted older adults' performance in complex tasks (Rossi et al., 2004; Vallesi et al., 2011; Diaz and Yalcinbas, 2021). Likewise, neuroimaging studies of aging shed light on older adults eliciting greater activation than younger adults in regions that have been implicated in attention, such as the superior parietal lobule (Diaz and Yalcinbas, 2021). Consistent with the hypothesis of compensation-related utilization of neural circuits (Reuter-Lorenz and Cappell, 2008), the brain used more cognitive resources at a time when older adults are underutilizing neural resources to achieve the levels of cognition similar to those of younger adults. Collectively, it may be a compensatory mechanism to aid processing to offset unimodal processing declines, which contribute to increasing audiovisual integration in older adults. Another possibility is based on the prior expectations of older adults. In addition, the benefits of using prior expectations in audiovisual integration are clear (Stein et al., 2014; Gau and Noppeney, 2016). According to Bayesian theories, participants shaped their prior expectations from the environmental properties and individual experience (Aitken et al., 2020; Plass and Brang, 2021). Possible differences in the experience used by the two age groups should be considered. Older adults have better crystal intelligence as they get older (from preexisting knowledge and experience) than younger adults (Zimprich et al., 2009). This means that older adults appear to be more susceptible to extensive experience and use it to synthesize audiovisual information. In addition, an additional possibility of improved audiovisual integration for older adults is the inhibition of irrelevant information in the auditory modalities. Using fMRI, Townsend et al. (2006) found that in a selective visual attention task, younger adults activated only brain areas involved in visual processing, whereas older adults transitioned to the activation of frontal and parietal areas and the sensory cortex. The authors suggested that these additional brain activations may reflect enhanced visual information and the suppression of auditory irrelevant information in older adults (Townsend et al., 2006). Lustig et al. also suggested that poor filtering for irrelevant stimuli may contribute to increased integration (Lustig et al., 2007). Collectively, the improvement of audiovisual integration was substantially more pronounced in older adults.

A key new finding in this study is that the interaction of age and attentional load was observed. At the time intervals of 140–210 ms, older adults have a greater audiovisual integration at the low-attentional load condition in comparison to younger adults. This phenomenon might be due to the attentional load acting on audiovisual integration. Attentional load theory suggests that if the load of the current task is low and its processing uses only part of the attentional resources, the excess attentional resources will automatically overflow to process the disruptive stimulus, thus producing an interference effect. In contrast, if the load of the current task is high and the limited attentional resources are exhausted, then what is irrelevant to the task cannot be processed. According to this, there are good reasons to believe that individuals will achieve optimal performance under the appropriate attention load. Taking into account the influence of a priori experiences and the unique compensatory mechanisms of the brain in older adults, it is no doubt that the improvement of audiovisual integration was substantially more pronounced in older adults in low-attentional load conditions. In summary, our results revealed that the age factor plays a key role in the interplay between integration and attentional load. However, the current studies could not identify whether the higher audiovisual integration in older adults at low-attentional load conditions is a compensation mechanism or results from appropriate levels of arousal, and future neuroimaging studies will need to clarify this issue.

## Conclusions

In this study, our findings confirmed that attentional load modulates the processing stages of audiovisual integration. There was weaker audiovisual integration under the no-attentional auditory load condition at the earlier processing stages; conversely, it was stronger in the late stages. Moreover, older adults benefit more from audiovisual integration than younger adults. These findings clarify the relationships between audiovisual integration and auditory attentional load in aging while offering positive evidence for the generality of load theory.

## Data availability statement

The original contributions presented in the study are included in the article/supplementary material, further inquiries can be directed to the corresponding author/s.

## Ethics statement

The studies involving human participants were reviewed and approved by the Ethics Committee of Hubei University. The patients/participants provided their written informed consent to participate in this study.

## Author contributions

Material preparation and data collection were performed by YR, ZL, and XY. Data analysis was performed by SL and YR. The first draft of the manuscript was written by SL and WY. All authors contributed to the study conception and design, commented on previous versions of the manuscript, and read and approved the final manuscript.

## Funding

This study was funded by the National Natural Science Foundation of China (31700973, 62103404, 31800932, and 32260198), the Humanity and Social Science Youth Foundation of Ministry of Education of China (16YJC190025 and 18XJC190003), Science and Technology Planning Project of Guizhou Province (QianKeHeJiChu-ZK [2021] General 120), Shenzhen Overseas Innovation Team Project (KQTD20180413181834876), Shenzhen Basic Research Program (JCYJ20210324101402008 and JCYJ20210324115810030), The CAS President's International Fellowship Initiative (2022VBA0031).

## Conflict of interest

The authors declare that the research was conducted in the absence of any commercial or financial relationships that could be construed as a potential conflict of interest.

## Publisher's note

All claims expressed in this article are solely those of the authors and do not necessarily represent those of their affiliated organizations, or those of the publisher, the editors and the reviewers. Any product that may be evaluated in this article, or claim that may be made by its manufacturer, is not guaranteed or endorsed by the publisher.
